# Risk Factors for Hemoptysis in Idiopathic and Hereditary Pulmonary Arterial Hypertension

**DOI:** 10.1371/journal.pone.0078132

**Published:** 2013-10-23

**Authors:** Darryl Tio, Edward Leter, Bart Boerrigter, Anco Boonstra, Anton Vonk-Noordegraaf, Harm Jan Bogaard

**Affiliations:** 1 Pulmonology Department, VU University Medical Center, Amsterdam, The Netherlands; 2 Clinical Genetics Department, VU University Medical Center, Amsterdam, The Netherlands; 3 Pulmonology Department, VU University Medical Center, Amsterdam, The Netherlands; Vanderbilt University Medical Center, United States of America

## Abstract

**Introduction:**

When hemoptysis complicates pulmonary arterial hypertension (PAH), it is assumed to result from bronchial artery hypertrophy. In heritable PAH, the most common mutation is in the BMPR2 gene, which regulates growth, differentiation and apoptosis of mesenchymal cells. The aim of this study is to determine the relationship in PAH between the occurrence of hemoptysis, and disease progression, bronchial artery hypertrophy, pulmonary artery dilation and BMPR2 mutations.

**Methods:**

129 IPAH patients underwent baseline pulmonary imaging (CT angio or MRI) and repeated right-sided heart catheterization. Gene mutations were assessed in a subset of patients.

**Results:**

Hemoptysis was associated with a greater presence of hypertrophic bronchial arteries and more rapid hemodynamic deterioration. The presence of a BMPR2 mutation did not predispose to the development of hemoptysis, but was associated with a greater number of hypertrophic bronchial arteries and a worse baseline hemodynamic profile.

**Conclusion:**

Hemoptysis in PAH is associated with bronchial artery hypertrophy and faster disease progression. Although the presence of a BMPR2 mutation did not correlate with a greater incidence of hemoptysis in our patient cohort, its association with worse hemodynamics and a trend of greater bronchial arterial hypertrophy may increase the risk of hemoptysis.

## Introduction

Pulmonary arterial hypertension (PAH) is a rare yet severe disease characterized by a progressive increase in pulmonary vascular resistance, right ventricular failure and premature death. Recent evidence from France suggests that the prevalence of PAH is about 15 per million [1].

Whereas PAH is primarily a disease of the pulmonary circulation, 75% of PAH patients show evidence of bronchial artery hypertrophy on CT analysis [2]. The number of dilated bronchial arteries increases with increasing PAH severity [3]. It is assumed that the presence of bronchial artery hypertrophy puts patients at risk for the subsequent development of hemoptysis. Hemoptysis was seen in 6% of PAH patients followed for 10 years in Poland [4]. Extrapolating recent survival data from France [5], the incidence of hemoptysis in the Polish cohort can be estimated at 1 episode per 137 patient years. Other than from hypertrophic bronchial arteries, hemoptysis in PAH may originate from pulmonary artery rupture. There have been several reports of hemoptysis in patients with aneurysms or pseudo-aneurysms of the pulmonary artery [6], [7], [8]. PAH is associated with progressive dilation of pulmonary arteries [9], [10] and together with an increased intraluminal pressure, pulmonary artery dilation may put patients at an increased risk of pulmonary artery rupture and subsequent hemoptysis.

Different subtypes of PAH are recognized, including idiopathic (IPAH) and heritable (HPAH) forms, and secondary PAH. HPAH is diagnosed in patients with proven germline mutations in genes associated with pulmonary hypertension as well as in familial cases with or without identified germline mutations [2]. The most prevalent genetic abnormality in HPAH is a mutation in the bone morphogenetic protein type 2 receptor gene (BMPR2). It has been estimated that 25% of all PAH patients have a BMPR2 mutation [3]. The BMPR2 gene is part of the transforming growth factor β signalling family and is involved in the regulation of growth, differentiation and apoptosis of mesenchymal and epithelial cells. The BMPR2 gene acts as a master switch mediating injury responses [9] and it is possible that loss of BMPR2 signalling reduces vessel stability. As a consequence, PAH patients who carry the BMPR2 mutation may have a reduced stability of pulmonary or bronchial vessels and may therefore be at an increased risk of rupture of blood vessels and subsequent hemoptysis.

In this study, we sought to assess hemoptysis rates in our patient cohort and to determine the relationship between the occurrence of hemoptysis, bronchial artery hypertrophy, pulmonary artery dilatation, and BMPR2 mutations in IPAH and HPAH.

Our aims were 1) To determine whether PAH patients who have suffered from hemoptysis exhibit a worse hemodynamic profile, more bronchial artery hypertrophy or more severe dilation of the main pulmonary artery; and 2) To explore whether a BMPR2 mutation puts PAH patients at a greater risk to develop bronchial hypertrophy or pulmonary artery dilation and subsequent hemoptysis.

## Methods

**Figure 1:**
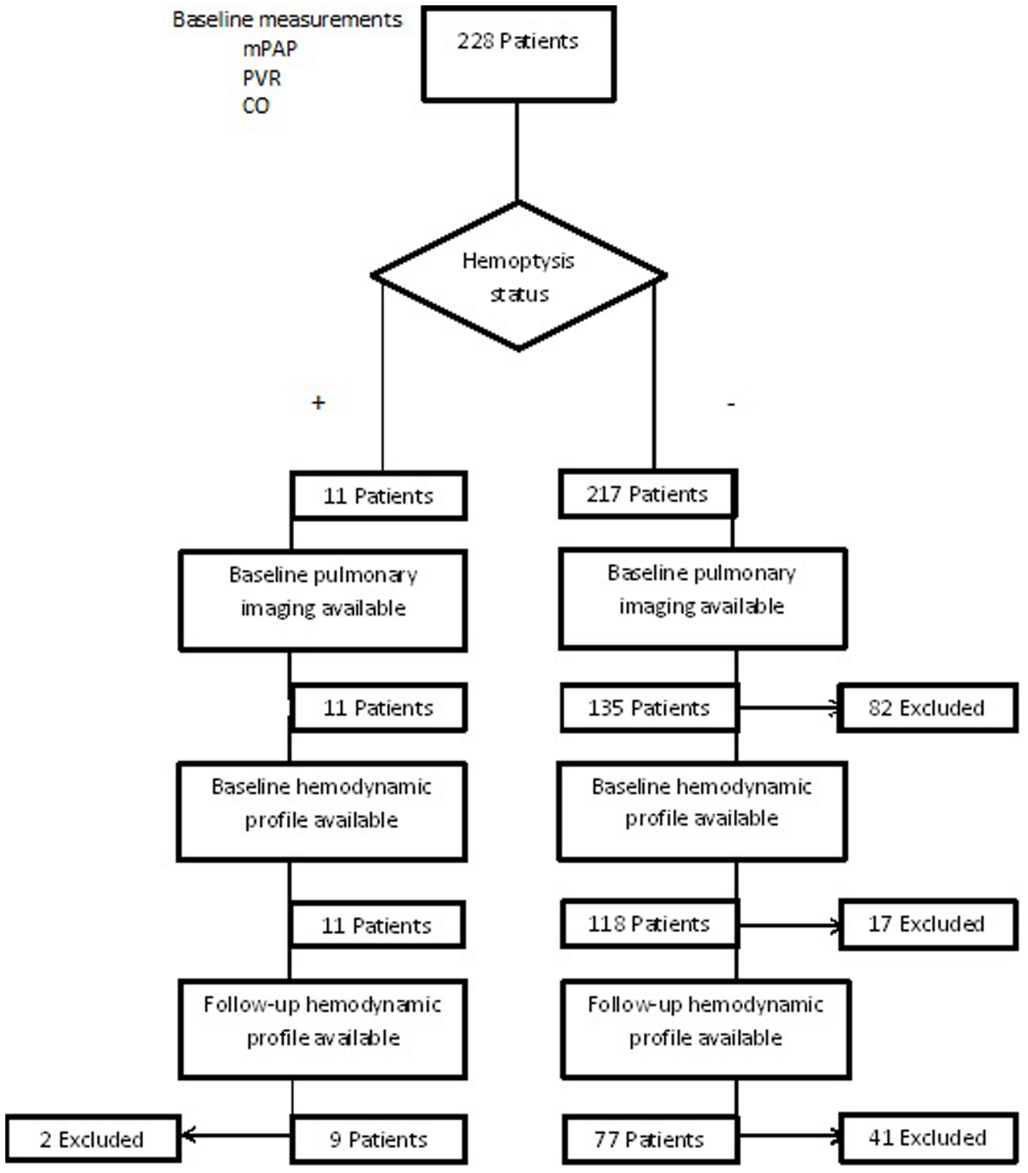
PAH Study cohort. Studies performed within 12 months of the original diagnosis were considered baseline data. Pulmonary imaging consisted of CT angiography and/or thoracic MRI.

This was a retrospective study based on the available medical records of 228 patients diagnosed with IPAH or HPAH (World Health Organization guidelines [11], between 1989 and 2011 at the VU University Medical Centre, Amsterdam, the Netherlands. Chronic Thrombo-embolic pulmonary hypertension (CTEPH) was excluded in all patients by performing ventilation perfusion scintigraphy The inclusion criteria for this study were 1) diagnosis of IPAH, 2) available baseline right heart catheterization (RHC) and Computed-Tomography angiography scan (CT angio) or Magnetic Resonance Imaging (MRI) performed within 12 months after initial diagnosis, 3) follow-up data available from diagnosis until death or the closing of the study. Patients were asked about occurrence of hemoptysis at every follow-up visit. Included in the study were self- reported episodes of hemoptysis and episodes occurring in the hospital. An episode was deemed significant if the hemoptysis consisted of 100 ml or more. In the cases of self-reported episodes, the patient was asked to describe the amount of blood in drinking cups, 100 ml being equal to ½ drinking cup. Out of the 228 patients, 11 patients experienced one or more episodes of hemoptysis (hemoptysis positive) and 217 patients were classified as hemoptysis negative. For 146 patients pulmonary imaging was available, and out of those patients, 129 had RHC data available. Median follow-up duration was 80.3±35.9 months. During follow-up, 5 patients died. Follow-up RHC data were available from 86 patients, including 9 patients with hemoptysis. Follow-up CT scans were performed in 81 patients (8 hemoptysis positive). Patients who did not undergo follow-up RHC or CT-scan/MRI were excluded from follow-up analysis (Figure 1).

This study was approved by the Institutional “Review board on Research Involving Human Projects” (IRB) of the VU University Medical Center, Amsterdam, The Netherlands. All patients were part of a longitudinal cohort study and gave written informed consent for the prospective collection of all their clinical data. According to the IRB approved protocol, no additional consent procedures are required for retrospective analyses of the database, which contains anonymous data only.

### Right heart catheterization

Hemodynamic assessment was performed with a 7-F balloon-tipped, flow directed Swan-Ganz catheter (131HF7, Baxter Healthcare Corp., Irvine, California) during continuous electrocardiography monitoring. PVR was calculated as: (mPAP –PCWP)/CO (mPAP is mean pulmonary artery pressure, PCWP is pulmonary capillary wedge pressure, and CO is cardiac output). [12].

### Pulmonary imaging

**Figure 2:**
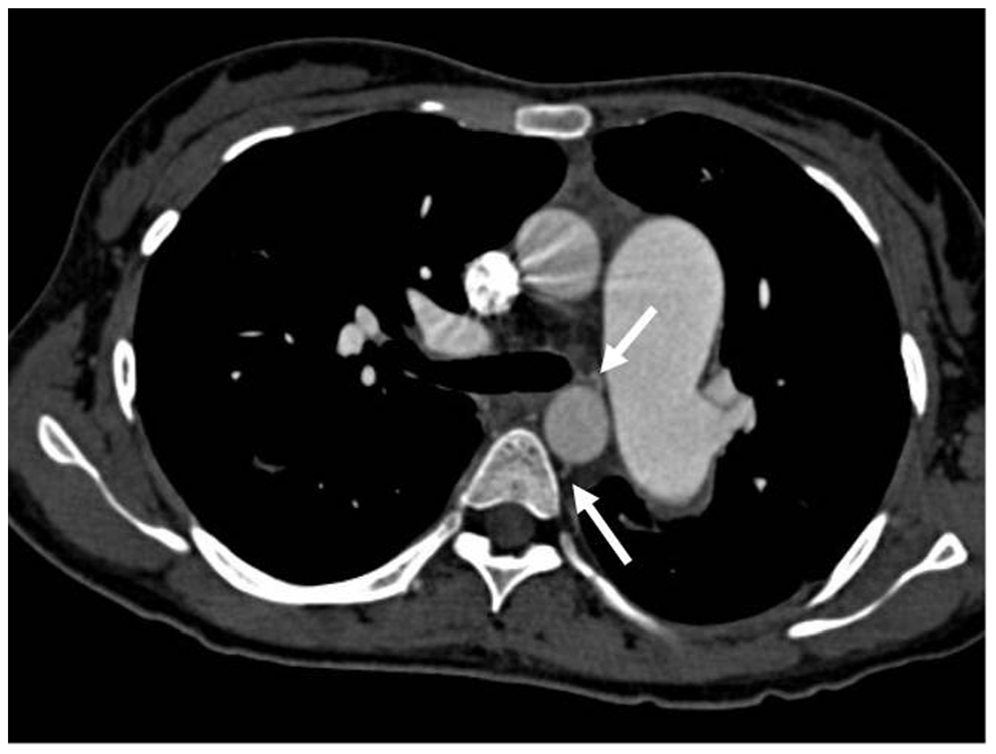
Shown is a CT angiography scan of a 23 year old iPAH patient with presence of bronchial artery hypertrophy. (upper arrow: left bronchial artery; lower arrow: right bronchial artery).

Bronchial artery hypertrophy was assessed on CT angiography and analysed by counting hypertrophied bronchial arteries on central CT slices (Figure 2). Bronchial arteries were considered hypertrophied if the diameter was >1.5 mm [3].

**Figure 3:**
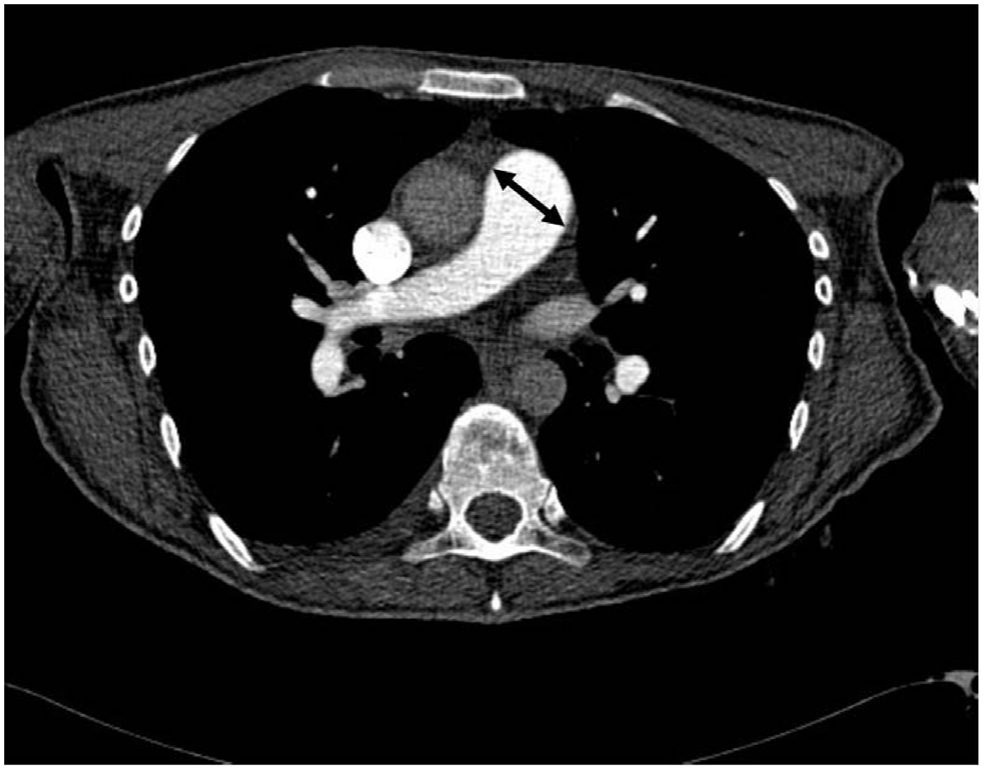
The pulmonary artery diameter was measured at the trunk of the pulmonary artery, on axial CT angiography slices using electronic callipers.

Pulmonary artery dimensions were analysed on a CT-scan or MRI scan, by measuring the diameter of the pulmonary artery using electronic callipers (Figure 3) [13], [14].

A sub-analysis was made consisting of all patients with available genetic data. Patients with a known BMPR2 status were examined for the occurrence of hemoptysis, bronchial artery hypertrophy and the degree of pulmonary artery dilation.

### Statistical analysis

Continuous variables were expressed as mean ± SD. Categorical variables were expressed as absolutes. To analyse differences between groups, the Students t-test or Mann Whitney-U test were used for parametric and non-parametric continuous variables, respectively. Categorical variables were analysed using a Chi-square test. Correlation coefficients were calculated using Pearsons correlation tests. P values <0.05 were considered statistically significant. Follow-up hemodynamic data were available in 86 patients and, a separate T-test was performed to test differences between hemoptysis positive and negative patients with regard to changes in mPAP and PVR. Growth of pulmonary arteries was analysed in 81 patients using a T-test. In the BMPR2 sub-cohort, follow-up RHC data was available in 44 patients and growth of pulmonary arteries could be determined in 38 patients.

## Results

Median follow up was 110.6±67.4 months for hemoptysis positive patients and 50.6±4.5 months for the negative patients (p = 0.01). Hemoptysis occurred after a mean follow up of 27.6±22.6 months. One year survival was 91% in this cohort.

### Baseline measurements

Table 1 summarizes the demographic and hemodynamic characteristics of 129 patients with baseline measurements. All baseline measurements were obtained in treatment-naïve PAH patients. The mean age was 50±15 years and 26% of the patients were male. 5 patients died during follow-up, 1 after an episode of hemoptysis. Patients had a total follow-up of 1259 years, 11 patients suffered an episode of hemoptysis resulting in 1 episode per 114 patient years. Patients who developed hemoptysis were significantly younger and had an earlier onset of disease.

**Table 1:** Baseline demographics and hemodynamics of the study population.

	Hemoptysis Positive (n = 11)	Hemoptysis Negative (n = 118)
Age (years)	42.9±12.8	57.3±17.9^¶^
Weight (Kg)	70.9±12.7	77.4±17.1
Height (Cm)	169±8	168±9
mPAP (mmHg)	60.6±14.8	52.6±15.4
PVR (dyn·s·cm^−5^)	863±385	855±495
CO	5.27±1.57	4.8±1.7
CI	2.98±1.01	2.60±0.96
6MWD (m)	449±101 (n = 5)	434±622 (n = 106)
6MWD (%pred.)	69.4±8.8 (n = 5)	76.0±9.1 (n = 106)

* =  p<0.001 versus Hemoptysis positive, #  =  p<0.01, ¶  =  p<0.05. Values are mean ± SD.

The presence of bronchial artery hypertrophy could be evaluated in 102 patients with CT angiography performed at baseline (see table 2). In the hemoptysis positive group, 1 patient had a single hypertrophied bronchial artery and 4 patients had 2 hypertrophied bronchial arteries at baseline (see table 2). In the group of hemoptysis negative patients with available CT scans, 26 patients had 1 hypertrophied bronchial artery while 5 patients had 2 hypertrophied bronchial arteries. There was a significant difference between the mean number of hypertrophied bronchial arteries per patient (p = 0.001). There was no difference in development of hemoptysis based on whether the left or right bronchial artery was hypertrophied (data not shown). The pulmonary artery diameter was not different between hemoptysis positive and negative patients (38.41±7.70 mm vs. 35.52±6.67 mm, p = 0.176).

**Table 2:** Bronchial artery hypertrophy in hemoptysis positive and negative patients with available baseline CT angiography scans.

	Hemoptysis Positive (n total = 11)	Hemoptysis Negative (n total = 91)
Hypertrophied bronchial arteries absent	6	60
1 hypertrophied bronchial artery present	1	26
2 hypertrophied bronchial arteries present	4	5
Total	11	91
Mean number of hypertrophied bronchial arteries per patient	0.82±0.98	0.40±0.59¶

¶ =  p<0.05 versus Hemoptysis positive.

### Follow-up measurements

Follow-up data was available for 86 patients (9 Hemoptysis positive). The average time between baseline- and follow-up measurements was 40.2±39 months and similar between hemoptysis groups. All follow-up measurements were done in PAH patients undergoing treatment, either with vasodilator monotherapy or different combinations of PAH specific therapies. Most patients used anti-coagulant therapy and all patients were on at least one pulmonary vasodilator drug. No differences in these therapies existed between groups and the use of pulmonary vasodilator drugs was not associated with an increased risk of hemoptysis (data not shown). Table 3 summarizes the follow-up demographic and hemodynamic characteristics of the study group. After follow-up, hemoptysis positive patients showed higher mPAP (65.4±11.3 mmHg, p = 0.002) and PVR (957±439, p = 0.017). No acute hemodynamic or clinical deterioration was noted in patients after an episode of hemoptysis.

**Table 3:** Baseline and follow-up demographics and hemodynamics of the study population in which follow-up data was available.

		Hemoptysis Positive (n = 9)	Hemoptysis Negative (n = 77)
Demographics at Baseline	Age (years)	42.9±12.8	52.6±16.7
	Weight (Kg)	70.9±12.7	74.8±16.4
	Height (Cm)	169±8	168±9
	mPAP (mmHg)	60.6±14.8	52.5±15.1
	PVR (dyn·s·cm^−5^)	863±385	967±505
	CO	5.27±1.57	4.66±1.63
	CI	2.98±1.01	2.57±0.91
	6MWD (m)	449±101 (n = 5)	502±771 (n = 67)
	6MWD (%pred.)	69.4±8.9 (n = 5)	72.0±18.4 (n = 67)
Data at Follow-up	Mean time between baseline and follow-up measurements (months)	48.1±37.6	41.1±34.1
	mPAP follow-up (mmHg)	65.4±10.9	46.1±13.7^#^
	PVR follow-up (dyn·s·cm^−5^)	957±439	620±341^¶^
	CO follow-up	5.57±2.49	5.41±1.52
	CI follow-up	1.93±0.43 (n = 2)	2.80±0.75 (n = 38)
	6MWD follow-up (m)	474±69 (n = 4)	446±135 (n = 64)
	6MWD (%pred.)	80.0±6.2 (n = 4)	80.1±21.4 (n = 64)

#  =  p<0.01 versus Hemoptysis positive, ¶  =  p<0.05. Values are mean ± SD.

Hemoptysis positive patients showed a trend of a larger total growth of the pulmonary artery during follow up (3.82±2.77 mm vs. 2.68±2.81, p = 0.149) and a trend of a faster growth per month (0.156±0.146 vs. 0.063±0.151, p = 0.107). Growth rates were not related to changes in mPAP in either group (data not shown).

### BMPR2 sub analysis

Genetic test results were available in 59 patients and hemodynamic data was available in 49 of these patients, and follow-up data in 44 patients. Table 4 summarizes baseline characteristics of the BMPR2 sub population. The mean age at diagnosis was 50.7±15.0 years. Of these patients, 23% were male. The median follow-up duration was 97.2±55.6 years and not different between BMPR2 positive and negative patients. Five patients died, and follow-up data was not available from 15 patients (including the deceased patients).

**Table 4:** Baseline demographics, hemodynamics and bronchial artery hypertrophy characteristics of the BMPR2 sub population analysis.

Demographics and hemodynamics at baseline		BMPR2 positive (n = 18)	BMPR2 Negative (n = 31)
	Age (years)	50.6±15.3	50.8±14.6
	Weight (Kg)	71.8±15.0	77.2±18.0
	Height (Cm)	165±5	171±10^¶^
	mPAP (mmHg)	56.4±14.3	55.5±14.0
	PVR (dyn·s·cm^−5^)	1107±644	782±354^#^
	CO	3.85±1.28	5.41±1.63^#^
	Cardiac index	2.18±0.78	2.91±0.95^¶^
	6MWD (m)	443±108 (n = 13)	396±141 (n = 23)
	6MWD (%pred)	78.2±18.1 (n = 13)	67.5±22.7 (n = 23)
	Mean number of hypertrophied bronchial arteries per patient	0.82±0.75	0.41±0.69

#  =  p<0.01, versus BMPR2 positive, ¶  =  p<0.05. Values are mean ± SD. CT angiograms were available from 11 BMPR2 positive patients and from 27 BMRP2 negative patients.

A BMPR2 mutation was not associated with a higher occurrence of hemoptysis. Two out of the 25 positive patients, had suffered from hemoptysis, and 4 out of 34 negative patients. (p = 0.738).

BMPR2 mutation positive patients showed a trend of more severe bronchial artery hypertrophy (p = 0.052) and a similar diameter of the pulmonary artery (35.01±9.19 vs. 35.57±6.06 mm, p = 0.115).

## Discussion

Here we show, in a representative cohort of iPAH and HPAP patients, a hemoptysis incidence of 1 episode per 114 patient years. We also show that the development of hemoptysis in PAH is associated with worse hemodynamics, more bronchial artery hypertrophy, a trend towards a greater pulmonary artery diameter and more rapid disease progression. The mean duration of follow-up was longer in hemoptysis positive patients, which suggests that the development of bronchial artery hypertrophy is slow and that a long duration of disease is required to develop hemoptysis.

In our relatively small cohort of tested patients, PAH patients carrying a BMPR2 mutation seemed equally prone to develop hemoptysis as BMPR2 negative patients, despite worse hemodynamics and a trend towards more bronchial artery hypertrophy.

Hemoptysis is a rare complication in patients with PAH. We found 1 case of hemoptysis per 114 patient years. This is in agreement with, our estimated hemoptysis incidence of 1 case of per 137 patient years using findings of Zylkowska *et al* [4], [5]. While we found bronchial artery hypertrophy in 45 of 102 (44%) PAH patients, its prevalence varied between 14%–75% in previous reports [2], [16], [17]. Bronchial artery hypertrophy seems to be more prevalent in CTEPH: between 73%–100% [16], [17]. Several authors have suggested that hypertrophied bronchial arteries predispose to hemoptysis in CTEPH [5], [18], the same is thought to hold true for PAH patients. Currently, the cause of bronchial artery hypertrophy in PAH is unknown. A possible explanation is tissue hypoxia to which the bronchial arteries react with hypertrophy. The association between the development of bronchial artery hypertrophy and hemoptysis suggests that hypertrophic bronchial arteries are frail and have a tendency to rupture. Whether a specific second hit is required for the development of hemoptysis in a patient with bronchial artery hypertrophy is unclear from the present study. It is possible that e.g. intercurrent lower airway infections render the bronchial mucosa and underlying vessels more vulnerable, thereby increasing the likelihood of hemoptysis.

**Figure 4:**
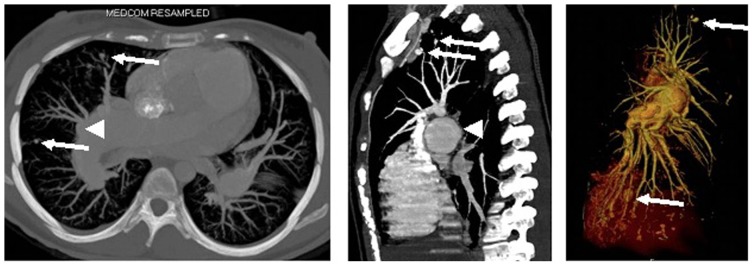
CT angiography from a 23 year old iPAH patient who died from massive hemoptysis. Panel A is a transverse section showing central pulmonary artery dilatation and small saccular aneurysms in more peripheral vessels (small arrows). Note the irregular borders of the central vessels. Panel B shows a coronal reconstruction with aneurysmatic changes to the main pulmonary artery (large arrow) and peripheral vessels (small arrows). Panel C is a 3 D reconstruction.

Although cases of hemoptysis from dilated pulmonary arteries have been reported [6], a relationship between growth rates of pulmonary arteries and hemoptysis was not previously considered. Boerrigter *et al*. found that dilatation of the pulmonary artery continues after the initial diagnosis, independently from changes in pulmonary artery pressure [9]. We confirmed these findings and also show that the growth of pulmonary arteries does not correlate with changes in hemodynamics. There have been reports of PAH patients suffering from aneurysms, dissections or even rupture of the pulmonary artery [6], [7], [8], [10], [20]. Senbaklavici *et al*. suggest that PAH strongly predisposes patients to dissection of the pulmonary artery, and that the false lumen tends to rupture, rather than developing a re-entry site, as is usual in aortic dissection [21]. We also found patients with aneurysms of the pulmonary artery (Figure 4) It can be argued that aneurysms, or dissections of the pulmonary artery have a greater risk of rupture due to the higher pressure in PAH. Although we found an association between pulmonary artery diameter and hemoptysis, it is unlikely that the majority of cases of hemoptysis in PAH are related to rupture of pulmonary arteries. The association between a greater pulmonary artery diameter and the occurrence of hemoptysis is rather explained by the fact that both pulmonary artery dilation and hemoptysis from hypertrophic bronchial arteries are independent indicators of the PAH severity.

Majka *et al*. have proposed that the normal function of the *BMPR2* gene is to act as a master switch mediating injury response. [19]. Reynolds *et al*. showed that experimental function loss of the BMPR2 gene leads to PAH [22]. Li *et al*. has shown that loss of *BMPR2* function contributes to abnormal pulmonary artery smooth muscle cell proliferation and predisposes to apoptosis of pulmonary endothelial cells [23]. We confirmed the results of Girerd *et al*., who showed that BMPR2 positive PAH patients have worse hemodynamics at diagnosis [15]. Surprisingly, worse hemodynamics did not translate to worse exercise performance in BMPR2 positive patients at baseline or follow-up. We did not find an association between BMPR2 status and hemoptysis, but this may be due to underpowering of our study, since we did find a trend of more bronchial artery hypertrophy in BMPR2 mutation positive patients. Whether bronchial artery hypertrophy (and perhaps, development of hemoptysis) in BMPR2 mutated patients, may result from loss of function of the BMPR2 gene or simply reflects a greater disease severity remains to be determined.

A limitation to this study was that some patients only underwent MRI scanning, not CT angiography. The incidence of bronchial artery hypertrophy was calculated for patients undergoing CT angiography only, while CT angiography and MRI were both used to measure pulmonary artery diameters. We found more hypertrophied bronchial arteries (43%) in our study, compared to findings of Endrys *et al*. (36%) and Remy-Jardin *et al*. (14%) in PAH patients, but these previous studies were done on only small cohorts of patients (11 and 14 patients, respectively), whereas we studied a total of 102 PAH patients. Another limitation was that not all patients were genetically tested. As mentioned before, only 6 patients suffering from hemoptysis had undergone genetic testing. This might have had an impact on the outcome of the results.

## Conclusion

Hemoptysis is a relatively rare complication of PAH, with one occurrence for every 114 patient years. A more progressive hemodynamic deterioration can lead to more bronchial artery hypertrophy and the subsequent development of hemoptysis. In a relatively small patient cohort, a BMPR2 mutation did not correlate with a greater prevalence of hemoptysis. However, due to its association with worse hemodynamics and a trend towards more bronchial artery hypertrophy, a BMPR2 mutation may increase the risk of developing hemoptysis.
